# Telomere Fragility and MiDAS: Managing the Gaps at the End of the Road

**DOI:** 10.3390/genes14020348

**Published:** 2023-01-29

**Authors:** Ryan P. Barnes, Sanjana A. Thosar, Patricia L. Opresko

**Affiliations:** 1 Department of Environmental and Occupational Health, School of Public Health, University of Pittsburgh, Pittsburgh, PA 15261, USA; 2 UPMC Hillman Cancer Center, University of Pittsburgh, 5117 Centre Avenue, Pittsburgh, PA 15213, USA; 3 Department of Pharmacology and Chemical Biology, School of Medicine, University of Pittsburgh, Pittsburgh, PA 15213, USA

**Keywords:** telomeres, replication stress, DNA replication, Mitotic DNA synthesis, telomere fragility

## Abstract

Telomeres present inherent difficulties to the DNA replication machinery due to their repetitive sequence content, formation of non-B DNA secondary structures, and the presence of the nucleo-protein t-loop. Especially in cancer cells, telomeres are hot spots for replication stress, which can result in a visible phenotype in metaphase cells termed “telomere fragility”. A mechanism cells employ to mitigate replication stress, including at telomeres, is DNA synthesis in mitosis (MiDAS). While these phenomena are both observed in mitotic cells, the relationship between them is poorly understood; however, a common link is DNA replication stress. In this review, we will summarize what is known to regulate telomere fragility and telomere MiDAS, paying special attention to the proteins which play a role in these telomere phenotypes.

## 1. Introduction

Telomeres are protective structures at the ends of linear chromosomes, which prevent erroneous activation of the DNA damage response (DDR). In humans and mice, they consist of 5′ TTAGGG 3′ repeats, which are bound by the shelterin complex, to form the protective t-loop [1]. This modifies the chromosome end into a structure resembling a recombination D-loop, thereby obscuring the 3′ single stranded DNA overhang end. While telomeres shorten in most somatic cells, cancer cells either upregulate telomerase or use a recombination-based mechanism (alternative lengthening of telomeres, ALT) to maintain telomere length. However, telomeres are inherently difficult to replicate for several reasons: (1) DNA polymerases are impeded by repetitive sequences [2], (2) the G-rich repeats can form stable G-quadruplex structures, which block replication [3], (3) the C-rich strand is transcribed and the resulting RNA can form DNA–RNA hybrids (R-loops), which stall replication [4,5] and (4) the t-loop itself needs to be dissolved for replication to proceed [6]. All of these facets which disrupt telomere replication induce what is known as DNA replication stress; the slowing or stalling of DNA replication forks. These inherent problems in replication make telomeres particularly sensitive to pharmacological or genetic sources of replication stress.

Telomeres, especially telomeres in ALT cells, are known to be hotspots of replication stress [7,8,9]. This ALT phenotype is primarily attributed to frequent loss of chromatin remodelers ATRX/DAAX, resulting in dysregulated chromatin, the accumulation of telomeric RNA (TERRA), and the insertion of variant telomeric repeats [10,11]. Replication stress is caused by non-B DNA structures, such as G-quadruplexes (G4) and RNA/DNA hybrids (R-loops), but also oncogene overexpression, drugs which inhibit DNA polymerases, and DNA lesions [12]. The response to replication stress is largely orchestrated by the ATR/Chk1 signaling pathway, which is activated by the accumulation of RPA-coated ssDNA [12]. This signaling acts both directly at replication forks, and also stalls the cell-cycle to allow for the completion of DNA replication. If a cell enters mitosis with incompletely replicated DNA, this can result in mitotic aberrations including segregation errors, chromatin bridges, and micronuclei [13,14]. When telomeres enter mitosis during replication stress, this can manifest as telomere fragility, which will be described in detail below.

A mechanism by which cells can repair under-replicated DNA is by performing DNA synthesis in mitosis (MiDAS). This mechanism is observed in cancer and non-transformed cells following drug or oncogene induced replication stress, and occurs frequently at common fragile sites (CFSs) and telomeres [15]. This post-replication repair pathway relies primarily on the replicative DNA polymerase delta (pol δ), which requires its sliding clamp PCNA and clamp loader RFC [16]. In general, MiDAS requires some remodeling of the ssDNA/ dsDNA junction, and occurs primarily in a conservative DNA synthesis pattern, which has led to a comparison to break-induced replication observed in yeast [17].

An outstanding question in the telomere field, however, is how telomere fragility and MiDAS relate to each other. They are both increased when cells experience replication stress, but few studies have examined these phenotypes simultaneously. In this review, we will summarize what is known about telomere fragility and MiDAS, including how they are detected and the factors which modulate their manifestation (Table 1). We will also speculate on the relationship between telomere fragility and mitotic DNA synthesis based on current evidence.

## 2. Telomere Fragility

### 2.1. Initial Discovery and Characterization

**Figure 1:**
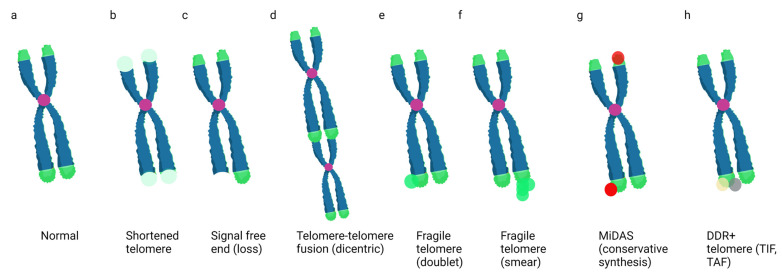
**Examples of Telomere Aberrations Detected on Metaphase Spreads**. Cartoons of a chromosome with normal telomere ends (**a**), shortened telomeres (light green) (**b**), a signal-free end or telomere loss (**c**), a telomere–telomere fusion or dicentric chromosome (**d**), fragile telomeres (**e**,**f**), mitotic DNA synthesis (**g**), and a telomere containing DNA-damage-response proteins such as γH2AX or 53BP1 (**h**). Green = telomeres; purple = centromeres; red = EdU foci; yellow and gray = DDR proteins. Created in BioRender.

Telomere fragility refers to a phenomenon in which multiple telomeric DNA foci are observed at the end of a chromatid by telomere PNA fluorescent in situ hybridization staining (FISH) (Figure 1). While not formally named until 2009, chromatid ends with multiple telomere foci were noted as early as 1999 and there have been several reports thereafter [41,65,66,67]. Both the de Lange and Blasco groups reported that TRF1-deleted mouse embryonic fibroblasts (MEFs) displayed increased chromatid ends with multiple telomere foci and termed this phenotype ‘telomere fragility’, due to the similarities with CFSs [19,65]. Importantly, both reports found conditions which induce unreplicated gaps or breaks at CFSs, namely aphidicolin (Aph)-treated and ATR-deficient cells, also increase telomere fragility. These results also suggested fragile telomeres may result from DNA replication problems. The de Lange lab confirmed this using single molecule analysis of replicated DNA (SMARD) to visualize telomere DNA replication fibers. In contrast to TRF2-deleted cells, loss of TRF1 reduced nascent telomere synthesis and increased fork stalling. Complementation of TRF1 knockout cells with a TRF1 mutant that cannot bind DNA (TRF1^ΔMYB^) could not rescue the fragile telomere phenotype, while loss of the N-terminal acidic domain could (TRF1^ΔAC^). These results suggested TRF1 binding to telomeric DNA is critical for suppressing fork stalling.

### 2.2. Telomere Fragility, DNA Unwinding, Fork Remodeling

In their 2009 publication, the de Lange lab observed telomere fragility in BLM helicase and RTEL1 helicase-deficient MEFs, but not in WRN helicase-deficient MEFs [19]. This increase was epistatic with TRF1 loss, and in a follow-up article they showed wild-type but not mutant TRF1 that abolishes BLM interaction rescued fragility [66]. One of the principal roles for BLM and RTEL1 at telomeres is the resolution of G-quadruplex (G4) secondary DNA structures. These structures form when there are tetrads of guanines in DNA, and are a natural impediment to replicative DNA polymerases. Reagents which stabilize G4s increase telomere fragility and disrupt replication, and these effects are additive with the loss of BLM or RTEL1. Surprisingly, loss of both BLM and RTEL1 causes an additive increase in telomere fragility, compared to single knockouts [6]. While this relationship has not been delineated, loss of BLM specifically increased fragility on the lagging strand [66]. However, BLM and RTEL1 unwind and translocate along DNA with opposite directionality [67], which may explain the need for both to prevent fragility.

Based on these data, one explanation for increased telomere fragility in TRF1-deficient cells is loss of BLM recruitment [66]. Further support for this model derives from analysis of BUB1/BUB3 deficient cells, which are important proteins in the mitotic checkpoint. These cells display increased telomere fragility, which is epistatic to the loss of BLM [61]. In this report, the Songyang lab found BUB1 knockdown reduced BLM and TRF1 interaction and that a kinase-dead BUB1 failed to rescue the telomere fragility phenotype. Moreover, the authors found BUB1 phosphorylated TRF1 in vitro, and a mutation of the predicted site (S296) abrogated BLM interaction, and its overexpression in cells increased telomere fragility. Although not related to BLM interaction, TRF1 was also reported to be phosphorylated on S404 by aurora kinase B [62]. Inhibition of this kinase increased telomere fragility, and overexpression of TRF1 S404A also increased fragility, and was epistatic with aurora kinase B inhibition. These studies indicate that helicase affinity for G4s is insufficient to prevent telomere fragility, and that proper telomere replication requires active recruitment of BLM helicase to the telomeres.

In addition to the helicase unwinding of secondary structures, stalled replication forks can be remodeled or reversed to promote replication fork restart. FANCM plays a prominent role in managing replication stress at ALT telomeres, and its loss exacerbates ALT phenotypes [50,51]. Over-expression of wild-type FANCM reduces telomere fragility in ALT cells, but mutants unable to interact with BLM or remodel the fork failed to rescue the phenotype, or exacerbated telomere fragility [50]. Key fork reversal enzymes have also been studied in the context of telomere fragility. In RTEL1-deficient cells with elevated telomere fragility, loss of PARP1 and ZRANB3 rescued the fragility phenotype, while ZRANB3 knockdown alone showed a modest increase in fragility in wild-type cells [29]. RTEL1 loss increases reversed forks, which impair replication when stabilized by aberrant telomerase binding. Thus, in the absence of RTEL1, preventing excessive fork reversal by PARP1 and ZRANB2 suppresses replication stress. Interestingly, in BLM-knockout cells which also have elevated telomere fragility, PARP inhibition increased fragility [25]. While loss of BLM and RTEL1 may generate different structures which are processed into fragile telomeres, PARP1 also has many roles in DNA repair, so these observations require further investigation. Finally, the loss of SMARCAL1 was also shown to increase telomere fragility [54]. Therefore, replication fork remodeling can promote or suppress telomere fragility depending on the extent of fork regression.

### 2.3. POT1 and CST Roles in Telomere Fragility

Aside from TRF1, other shelterin and telomere binding factors have been shown to suppress telomere fragility. POT1 binds to the telomeric single-stranded overhang to prevent inappropriate ATR kinase activation by ssDNA which signals replication stress [1]. The Denchi and Sfeir labs showed that Pot1a loss in mouse thymocytes increased telomere fragility, and that expression of POT1 mutants associated with cutaneous T cell lymphoma in human and mouse cells impaired POT1 function and increased telomere fragility [32]. Expression of these POT1 mutants in MEFs also increased fork stalling in telomeres, as visualized by the SMARD assay, similar to TRF1 loss.

STN1, CTC, and TEN1 form the CST complex which also promotes DNA replication, especially at telomeres [68]. Loss of either CTC1 or STN1 increases telomere fragility in human cells, and this is related to their roles in promoting c-strand fill in and suppressing telomere G4s [22,27,53]. Cancer-associated POT1 mutations lead to reduced CST association with telomeres, and are epistatic with CST loss in increasing telomere fragility, suggesting POT1 and CST prevent fragility in the same pathway, but this requires further study [32].

Work from the Boulton lab revealed that TRF2 interacts with, and facilitates recruitment of, RTEL1 to the telomeres [69]. However, while a TRF2 mutant that abolishes this interaction prevented RTEL1 function in unwinding T-loops, it did not prevent RTEL1 function in suppressing telomere fragility. Therefore, TRF1 and POT1 are the primary shelterin factors that prevent telomere fragility.

### 2.4. Excision-Repair Proteins, Oxidative DNA Damage and Telomere Fragility

Several studies demonstrated a role for DNA excision-repair proteins in suppressing telomere fragility. Earlier work from the Blasco lab showed the knockout of nucleotide excision-repair (NER) protein XPC, in mouse embryonic fibroblasts, led to elevated telomere fragility, which was reduced when cells were grown at 3% oxygen compared to 20% oxygen [28]. Wild-type MEFs and human cells also showed reduced fragility when cultured at 3% oxygen [27], implicating oxidative DNA damage in fragility. Consistent with these results, targeted oxidative damage to telomeres in the form of 8-oxo-guanine (8oxoG) also increases telomere fragility [23]. Loss of the base excision-repair (BER) enzyme OGG1 dramatically elevated telomere fragility in cancer cells after chronic 8oxoG formation at telomeres [24]. Finally, there is some evidence for the role mismatch repair (MMR) proteins in telomere fragility in ALT cells [58,59]. The O’Sullivan group showed the loss of MSH6 increased telomere fragility, while the Peña-Diaz group found MSH3 loss, and not MSH6 loss, increased fragility. Curiously both proteins require MSH2 to function in the MMR pathway, but loss of MSH2 also did not increase fragility.

More recent work indicates that the TFIIH transcription complex suppresses telomere fragility through an interaction with TRF1, but that this is unrelated to its role in NER [60]. While TFIIH, along with XPB and XPD helicases, has a well-established role in duplex melting at DNA lesions in nucleotide excision repair (NER), the de Lange lab found depletion of other NER factors did not increase telomere fragility in this study. This novel non-canonical role for the TFIIH complex at telomeres may be related to the ability of the associated helicases to resolve secondary DNA structures.

## 3. Mitotic DNA Synthesis (MiDAS)

### 3.1. Initial Discovery and Characterization

While the bulk of DNA replication occurs in S-phase, DNA repair synthesis can occur in all phases of the cell cycle, including mitosis. The relatively recent discovery of DNA synthesis in mitosis, was first reported in human cells experiencing Aph-induced replication stress in the absence of DNA polymerase (Pol) eta [70]. This was followed by seminal papers from the Hickson lab which characterized DNA synthesis in mitosis termed MiDAS. They showed replication-stress-induced MiDAS frequently occurs in a conservative DNA synthesis pattern in prophase, and depends on Pol δ, MUS81, and Rad52 [15,17]. Consistent with MiDAS occurring after normal S-phase replication, the replisome disassembly factor TRAIP, which targets the CMG helicase for degradation, is required for MiDAS in cancer cells, indicating that the replicative helicase needs to be removed for this post-replication repair pathway [71,72]. While most MiDAS studies have utilized cancer and transformed cell lines, normal cells can also employ MiDAS following replication stress [73]. Interestingly, however, in non-cancerous cells, MiDAS is dependent on FANCD2 but not RAD52 [73]. In cancer cells FANCD2 usually co-localizes with mitotic EdU foci, and breaks at CFSs, so its relevance to MiDAS is not surprising [15,74]. This highlights that the factors required for MiDAS have some contextual dependence, which we will revisit in the telomere MiDAS section of this review.

### 3.2. Telomere MiDAS

**Figure 2:**
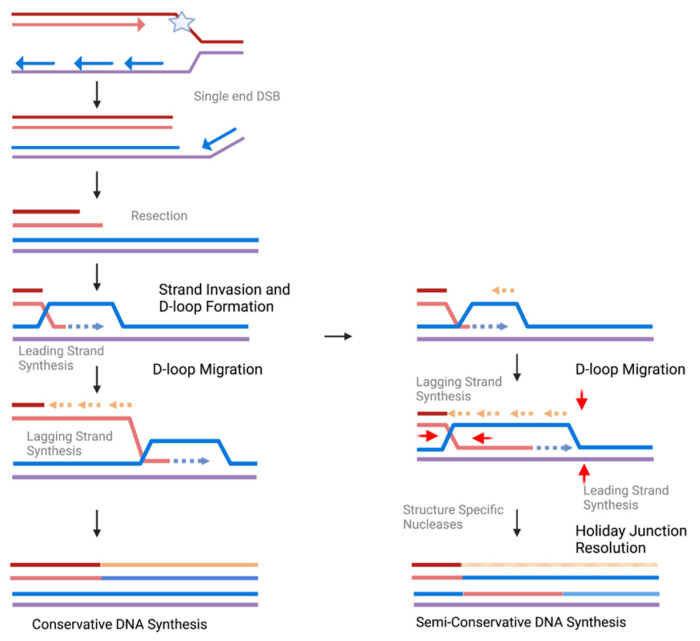
**Schematic of DNA Synthesis During MiDAS**. When a replication fork collapses following replication stress (star), a single-ended double-strand break forms. After resection, the 3′ overhang of the recipient strands (red/orange) can invade the donor stands (blue/purple). After D-loop formation, if lagging-strand synthesis takes place on the invading stand, no new synthesis will occur for the donor strand, resulting in conservative DNA repair synthesis. If instead lagging-strand synthesis occurs on the extruded donor D-loop strand, following D-loop migration structure specific nucleases (red arrows) will resolve the holiday junction. This results in new synthesis for both the donor and receipt and is semi-conservative DNA repair synthesis. Created in BioRender.

The first studies focusing on telomere-specific MiDAS were conducted by the Shay and Hickson labs [20,21]. Both groups visualized telomere MiDAS by pulsing mitotic cells following G2 arrest with the nucleotide analog 5-ethynyl-2-deoxyuridine (EdU) and prepared metaphase chromosome spreads. They found cancer cells display both spontaneous and replication-stress-induced MiDAS at telomeres, even though the replication stress is not telomere-specific. This telomere MiDAS was observed with Aph treatment, oncogene over-expression, G4 stabilizer ligands, and knockdown of replication fork protection proteins, as well as RNAseH1 [21]. Importantly, both labs found telomere MiDAS occurs frequently on a single chromatid end, representing a conservative form of DNA synthesis. Conservative synthesis in DNA repair is thought to arise from break-induced DNA replication (BIR) [75]. In contrast to normal replication, BIR involves strand invasion from the 3′ end of one chromatid into another, generating a D-loop which can migrate and allow for leading, and then lagging, strand synthesis. Since the lagging strand utilizes the newly extended invading leading strand as a template, the other strand of the invaded chromatid is not copied, resulting in conservative replication (Figure 2). Studies in yeast found BIR requires the POLD3 subunit of Pol δ [76], and this is also true for genome-wide MiDAS in human cells [15]. This led to the proposal that MiDAS in general, and at telomeres, is a form of BIR.

While BIR may respond to collapsed replication forks which become a single-ended DSB, these breaks can also occur through processing by nucleases, as seen with the expression, or instability, of common fragile sites [77]. Consistent with genome-wide MiDAS in cancer cells, telomere MiDAS is RAD52- and SLX4-dependent, but in contrast, is MUS81-independent [20]. SLX4 serves as a scaffold for multiple nucleases including SLX1 and XPF, suggesting the activity of another nuclease is needed for telomere MiDAS. Indeed, the inhibition of the MRE11 nuclease significantly reduced telomere MiDAS in cells lacking tipin, a replication fork protection factor, and cells experiencing telomere-specific oxidative stress [21,24]. Together, these studies show MiDAS in human cells requires factors consistent with BIR, and suggest that BIR-like processes can occur at telomeres via MiDAS.

### 3.3. BIR and MiDAS at ALT Telomeres

BIR was characterized as a mechanism by which telomerase-deficient yeast strains maintain their telomeres during ALT [78,79]. This occurred in Rad51-dependent and Rad51-independent pathways, but is fully Rad52-dependent, consistent with MiDAS. Human cancer cells that use ALT are characterized by the presence of PML-associated bodies (APBs), heterogeneous telomere lengths, and extra-chromosomal DNA. ALT telomeres experience elevated replication stress and DNA damage, and as such, they require DNA repair proteins such as helicases (BLM and FANCM) [80,81], recombination factors (RAD51AP1 and BRCA1) [47,82], nucleases and damage sensors (SLX4/1 and MRN) [83,84], specialized DNA polymerases (Pol eta) [55], and other repair proteins to stably maintain them. Both the Hickson and Shay groups observed significantly higher telomere MiDAS in ALT cells compared to telomerase-positive cells spontaneously or following induced replication stress [20,21].

BIR was also invoked to explain how ALT telomeres are maintained prior to the focus on telomere MiDAS. The Greenberg group showed that the production of enzymatic breaks at telomeres with the FokI-TRF1 tool led to a significant increase in telomere DNA synthesis, which was dependent on Pol δ for synthesis of both the C- and G-rich strands (POLD1 and POLD3) [16]. By arresting cells in G2 with a cyclin dependent kinase 1 inhibitor (CDK1i), they found this break-induced telomere synthesis (BITS) can occur outside of S-phase, even in the absence of FokI-TRF1, and was dependent on Rad52 [34]. While both ALT and telomerase-positive cells are capable of BITS, only ALT cells display appreciable levels of spontaneous telomere DNA synthesis outside of S-phase, as monitored by BrdU incorporation and immunofluorescence (IF) microscopy. This group later showed BITS can occur in nocodazole-arrested mitotic cells, and was dependent on POLD3 [34]. In these experiments, cells are arrested at metaphase, and then collected before FokI-TRF1 breaks are induced. Therefore, while this demonstrated BITS can occur in mitotic cells, it may be distinct from replication-stress-induced MiDAS, which does not occur after prophase [15]. Indeed, while telomere MiDAS requires SLX4, the Greenberg group found spontaneous ALT G2 DNA synthesis and BITS are independent of SLX4, highlighting that the two phenomena, while similar, may be distinct [34].

### 3.4. Shelterin, SUMO, and MiDAS

While the telomere binding proteins TRF1, TRF2, and POT1 have been known to regulate the DDR at telomeres, there is also evidence that they regulate MiDAS. In MEFs with conditional TRF1 deletion, loss of TRF1 resulted in conservative MiDAS [30]. While this was POLD3-dependent, it was independent of SMC5. SMC5 and SMC6 help establish cohesion, and respond to stalled replication forks and DSBs. SMC5/6 also have SUMO ligase activity which is required for their function in sister chromatid cohesion, and interestingly the Shay group showed these proteins were required for telomere MiDAS in ALT cells [21]. The differences may be due to the fact ALT telomeres are heavily reliant on SUMO for APB formation and telomere integrity [85].

TRF1 and TRF2 are SUMOylated, and this is critical for ALT [85]. The Shay group showed mutation of TRF2 SUMOylation sites reduced telomere MiDAS, and over-expression of a TRF2 SUMO fusion protein increased MiDAS and G2 telomere synthesis [31]. In the same report, they found over-expression of wild-type and a mutant BLM, which cannot be SUMOylated, increased telomere MiDAS, while a helicase-dead and Sumo-interacting motif (SIM) mutant BLM had no increase in MiDAS. Although the necessity of BLM for telomere MiDAS has yet to be tested by depletion, loss of BLM does eliminate spontaneous G2 ALT DNA synthesis [34]. Since BLM is known to interact with TRF1 and TRF2 [80], it is tempting to speculate that the requirement of BLM’s SIM for promoting telomere MiDAS is due to SUMOylated TRF2/1.

The mutation of shelterin POT1 single-strand binding domain (POT1-ΔOB) also increased telomere MiDAS in both cancer and p53-deleted RPE-1 cells [33]. This study also found knockdown of the MiDAS factors POLD3 and SMC2 reduced the viability of POT1-ΔOB cells, although the study did not directly test for MiDAS in those conditions. POT1-ΔOB is recruited to telomeres, but since it cannot bind ssDNA, telomeres are deprotected leading to a DDR, which is believed to be due to ATR activation [32,33,86]. ATR inhibition had been shown by the Hickson group to increase MiDAS following Aph, while the Shay group had observed a decrease in telomere MiDAS with ATRi [17,21], which would be consistent with the POT1-ΔOB result. The differences observed here may be due to methodology. The Shay group added ATRi during mitosis, while the Hickson group added ATRi to S-phase cells in the presence of the Cdk1i, but not in mitosis. Therefore, in the Hickson lab experiment ATR was likely active in mitosis, but its inhibition caused replication stress prior to G2, requiring MiDAS for repair. However, this remains to be directly tested, especially since MUS81 has differential requirements for general and telomere-specific MiDAS.

## 4. Replication Stress Enhances Telomere Fragility and MiDAS

Previous studies have consistently shown that reagents or genetic depletions which induce replication stress increase both telomere fragility and MiDAS (Table 1). In this section we will try to summarize the literature of genetic and pharmacological conditions in which both phenomena were observed.

### 4.1. TERRA, RNA/DNA Hybrids, and G4s

TERRA is a long non-coding RNA transcribed from the C-rich leading strand of telomeres which can act at the transcribed telomere *in cis* or associate with other telomeres *in trans* [87]. TERRA is proposed to play several positive roles at telomeres, including telomerase regulation [88], heterochromatin formation [89], and the telomere DDR [90]. To facilitate telomere replication, TERRA levels are high in G1/early S-phase, which allows for RPA binding to telomere ssDNA due to TERRA sequestering hnRNP1A. In mid-S/G2-phases, TERRA levels decrease, which releases hnRNP1A, allowing it to displace RPA and promote POT1 binding [91,92]. Loss of hnRNPA1 increases telomere fragility, suggesting telomere replication is impaired due to POT1 dysregulation, whose downregulation is known to increase telomere fragility and MiDAS (see above) [43]. Further support for a DNA-replication and TERRA-regulation connection derives from the observation that flap endonuclease 1 (FEN1) downregulation specifically increases leading-strand telomere fragility, which is the strand TERRA is transcribed from [57]. While not demonstrated directly, it is tempting to speculate that FEN1 degradation of TERRA during S-phase promotes the RPA-POT1 switch after DNA replication.

TERRA localization to telomeres promotes R-loop formation, and is regulated by other RNA binding proteins and nucleases, as well as helicases. TERRA foci are suppressed by RNA binding proteins UPF1 and SMG1, as well as NONO and SFPQ, and their loss in cells increases telomere aberrations and fragility [4,42]. Downregulation of RNAseH1, which specifically digests RNA/DNA hybrids, increases both telomere fragility and telomere MiDAS [21], while over-expression of RNAseH1 specifically reduces leading-strand fragile telomeres [93].

RTEL1 is known to suppress G4 structures, but its loss also increases RNA/DNA hybrids in cells [35]. While this increase was not shown for telomeres specifically, loss of RTEL1 reduced Aph-induced telomere MiDAS, resulting in an increase in chromatin bridges containing telomeric DNA. Chromatin bridges between daughter cells are believed to arise from incomplete DNA synthesis at DNA replication intermediates.

### 4.2. Stalled Fork Processing: HR, Nucleases, and Protection

Earlier studies in yeast and mammalian cells proposed that telomeres were sites of late DNA replication, and required DNA damage signaling and repair proteins to ensure complete synthesis [94,95]. Various HR and replication fork protection (FP) factors, including RAD51, RAD52, and BRCA1/2, were shown to associate with telomeres late into the cell cycle and BRCA2 recruitment was in part ATR-dependent [46,94]. As described above, telomere fragility and MiDAS are increased when ATR is inhibited, and in agreement, loss of HR and FP has a similar effect.

Loss of HR factors BRCA2 and RAD51 increases both telomere fragility and MiDAS [21,38,45]. While telomeric MiDAS has not been explored in RAD51AP1 or BRCA1 deficient cells, interestingly, both proteins have reported discrepancies concerning fragility. In U2OS ALT cells, BRCA1 loss increases fragility but has no effect in MEFs, while loss of RAD51AP1 increased fragility in one report, and decreased it in another, both in U2OS cells [25,44,45,47]. The difference for RAD51AP1 may lie in siRNA vs CRISPR/Cas9 knockout, while the BRCA1 difference may be due to human ALT versus murine telomerase status.

Several nucleases have been tested for their effect on telomere stability. SLX4 knockout MEFs and U2OS cells have increased telomere fragility [34,39]. However, SLX4 and SLX1 depletion reduced telomere fragility caused by BLM knockout [25]. Loss of MRE11, of the MRN complex, was shown to increase telomere fragility modestly in MEFs from generation 4 (Gen4) *Terc* knockout mice [38]. The same report showed that in these Gen4 MEFs, loss of BRCA2 dramatically elevates telomere fragility, but this is suppressed with MRE11 knockdown. Collectively, these studies suggest that when telomere replication stress is increased by lack of BRCA2 or BLM, nuclease cleavage of the replication intermediates is involved in the observed fragility. This is reminiscent of the relationship between MUS81 and CFSs [77]. When cells are treated with Aph, loss of MUS81 prevents CFS breaks due to MUS81′s function in cleaving the under-replicated DNA intermediates. Consistent with this, loss of SLX4 and MRE11 reduces telomere MiDAS in cells experiencing replication stress [20,21], suggesting the processing of stalled replication intermediates is required for DNA synthesis to restart.

Timeless, tipin, and claspin are members of the replication fork protection complex (FPC) which mediates Chk1 activation during replication stress [96]. Consistent with this role, loss of timeless increases telomere fragility, and loss of tipin or timeless increases telomere MiDAS [21,52]. Finally, FANCD2 has a well-established role in genome and CFS stability, and in marking sites of MiDAS genome-wide [15,97]. Loss of FANCD2 elevates telomere fragility in primary cells from Fanconi anemia patients, and when depleted by siRNA in ALT cells [48,49]. While only one ALT and telomerase cancer cell line were compared for telomere fragility in this study, the authors also show FANCD2 loss results in telomere hyper-extension in a BLM-dependent manner in ALT cells [48]. FANCD2 loss also reduced POLD3 and PCNA recruitment to telomeres in G2-phase, consistent with its role in supporting MiDAS [73,98]. These observations suggest that loss of FANCD2 results in aberrant telomere replication and reduced MiDAS, leading to under-replicated DNA persisting into metaphase.

## 5. Telomere Replication Stress as a Therapeutic Opportunity

Given that telomere maintenance is a hallmark of cancer, elucidating indicators and mechanisms of replication stress at telomeres has important therapeutic implications for treating cancer. The impairment of telomere replication in cancer cells through G4 ligands, oxidative damage, ATR kinase inhibitors and other factors listed in Table 1 could deplete telomeres and halt cellular proliferation. ALT cells may be especially sensitive to telomere replication perturbations, since they show both more telomere fragility and MiDAS compared to telomerase-positive cancer cells [21]. ALT telomere maintenance is essentially a response to the elevated levels of replication stress at telomeres. ALT cancers rely on replication stress to direct DNA repair pathways to the telomeres to facilitate homology-directed extension of telomeres. However, this characteristic makes ALT cancers highly sensitive to further replication insult, thus requiring factors that alleviate this excess replication stress particularly at the telomeres (for recent reviews see [8,99]). ALT cancers harbor positive regulators, which are sources of replication stress and often increase telomere fragility and MiDAS, that are tightly balanced with negative ALT regulators which suppress replication stress (see Table 1 and [8,9]). These positive and negative ALT regulators finely balance telomeric replication stress resulting in telomere maintenance and cell viability. Disrupting this balance can thus promote toxic levels of telomeric damage resulting in unfavorable cellular outcomes. This provides a broad, but precision based therapeutic opportunity to target ALT cancers specifically by modulating replication stress.

Proteins that are involved in relieving telomere-specific replication stress as well as those involved in the replication stress response pathway are being investigated as therapeutic targets in ALT cancers. These include ATR and ATM inhibitors, such as the ATM inhibitor AZD0156 in ALT positive neuroblastoma [100]. Some studies have demonstrated that ALT cancers may be more sensitive to ATR inhibition compared to telomerase positive cancers [101,102]. While this finding has been challenged by contradictory studies [100,103,104], ATR inhibition in conjunction with replication-stress-inducing therapies, such as chemotherapies and PARP inhibition, could further prove to be beneficial against ALT tumors. In 2019 the Pickett lab showed that ALT cells are hypersensitive to replication stress caused by FANCM depletion [50], compared to telomerase-positive cancer cells, highlighting FANCM as an ALT therapeutic target. Lastly, the finding that telomeric MiDAS is RAD52-dependent in ALT cells (Table 1) provides opportunities for targeting RAD52 in ALT tumors that harbor elevated amounts of telomeric replication stress.

## 6. How Are Telomere Fragility and MiDAS Connected?

The mechanistic relationship between telomere fragility and MiDAS remains to be fully dissected. Deciphering the connection is challenging because while post-replicative DNA synthesis can be detected as overlapping EdU and telomeric DNA staining in G2 or mitotic cells or on metaphase chromosomes, telomere fragility can only be detected on metaphase chromosomes by telomere FISH. In this section we propose several scenarios that may explain the positive association between telomere fragility and telomere MiDAS, including possibilities that these events are linked and/or independent. We also speculate on the mechanistic and temporal relationship between the two, based on evidence that fragile telomeres may arise from BIR or incomplete DNA synthesis.

Links between MiDAS and telomere fragility have been studied by examining whether metaphase telomeric ends showing EdU incorporation (MiDAS) also appear as fragile. The Hickson group reported that spontaneous MiDAS in ALT cells occurred more frequently at telomeres showing the fragile phenotype; however, the opposite was true after Aph treatment [20]. This led to the conclusion that replication-stress-induced telomere fragility and MiDAS phenotypes arise from independent events. Another possibility is that fragile telomeres may fail to co-localize with EdU incorporation if the replication track that leads to telomere fragility is too small to cause the aberrant chromatinization that may manifest as doublet or smeared signals at the chromatid end. However, given the large overlap of causes and regulators of these two phenomena, further investigation is warranted.

More recent work provides evidence that telomere fragility arises from BIR events. The de Lange lab reported that fragile telomeres in BLM-deficient cells are detected by standard telomere FISH, but not by CO-FISH (when quantifying leading and lagging strands together) which involves degradation of the newly synthesized strands [25]. Therefore, they concluded that telomere fragility in BLM-deficient cells involves conservative BIR. Consistent with BIR, the appearance of telomere fragility in BLM-deficient cells required POLD3, POLD4, and SLX1/SLX4 nuclease, but not MUS81 or proteins that promote fork reversal. However, they proposed that BIR likely occurred in S-phase because they observed telomeric DDR foci primarily in S-phase cells, and no EdU incorporation (MiDAS) at metaphase telomeres showing the fragile phenotype. This led to a model in which unreplicated gaps at G4 may be cleaved by SLX4/SLX1 to form a DSB which initiates BIR. The implication of this study is that BIR in S-phase may manifest as fragile telomeres on metaphase chromosomes, while links to BIR-like activity during mitosis (i.e., MiDAS) were less apparent. If BIR was successful prior to mitosis, a telomere would likely not appear fragile at metaphase if the fragility was caused by under-replicated DNA.

**Figure 3:**
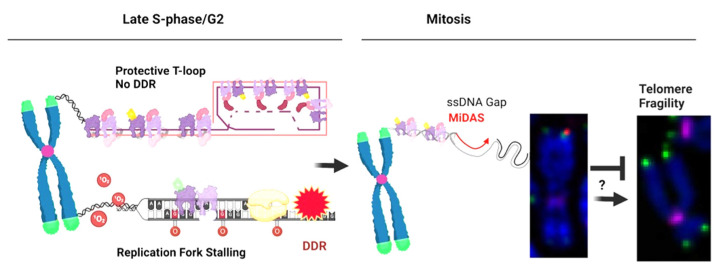
**Speculative Model of the Relationship between Telomere Fragility and MiDAS**. A normal chromatid end is folded into the protective T-loop structure by shelterin, which prevents DDR and chromosome fusions. When a replication fork stalls, by 8-oxo-guanine for example, this triggers a DDR. If replication is not completed by the end of G2, the under-replicated gap will persist when the cell enters mitosis. In order to fill the gap and maintain genome stability, cells can conduct telomere MiDAS in prophase. If MiDAS fails or is incomplete, under-replicated gaps will likely lead to mitotic segregation errors in the cell. When arrested in metaphase, however, this altered telomere structure can be observed with FISH, resulting in doublets or smeared signals. Created in BioRender.

Another possibility is that fragile telomeres may manifest from stretches of ssDNA (or gaps) due to the presence of unresolved DNA replication and/or recombination intermediates, caused by failures to complete DNA synthesis in G2 or mitosis (Figure 3). Consistent with this, previous reports have proposed that telomere fragility may arise from aberrant chromatinization or condensation due to the presence of unreplicated DNA [19,105]. We propose telomere MiDAS may also arise from DNA synthesis in ssDNA during gap filling, as well as BIR. Consistent with this, numerous studies have reported that ssDNA gaps arise from unrestrained DNA replication, failures in repriming, or Okazaki fragment processing, and can be responsible for cellular toxicity caused by the PARPi treatment of BRCA deficient cells (reviewed in [106]). As shown in Table 1, BRCA1, BRCA2, and PARPi also modulate telomere MiDAS depending on the context, and therefore, this requires further exploration.

In summary, while telomere fragility and telomere MiDAS are both elevated in ALT cells and increase in response to replication stress, more work is required to determine why these events are correlated, and how mechanistically they may be linked. Key to advancing our understanding will be deciphering the molecular structure of fragile telomeres, and the mechanism(s) that initiate and carry out post-replicative DNA synthesis at telomeres. Given that telomere maintenance is required for cancer cell immortalization, understanding how replicative stress at telomeres is managed, especially in ALT cancers, will be important for developing cancer therapeutics that target telomeres.

## Figures and Tables

**Table 1:** List of experimental manipulations and genetic depletions that impact telomere fragility, telomere mitotic DNA synthesis, spontaneous telomere DNA synthesis in G2 (ALT), and FokI-TRF1 induced DNA synthesis in G2/M.

Effects of Protein Depletion or Reagent Addition *				
	Fragility	Mitotic DNA Synthesis	Spontaneous G2 Synthesis	BITS (FOKI-TRF1)
**Reagent**				
ATRi/ATR-deficiency	Increase [18,19]	Increase [20]		Increase [16]
ATMi		No Change [21]		
Aphidicolin	Increase [19]	Increase [20]		
G4 ligand	Increase ^b^ [21,22]	Increase [21]		
FAP-TRF1 8oxoG	Increase [23]	Increase [23,24]		
FokI-TRF1 break	Increase [25]			Increase [16]
Oncogene OE ^a^	Increase [21,26]	Increase [21]		
Oxidative stress	Increase [27,28]			
PARPi	Decrease ^g^ [29] Increase ^c^ [25]			
**Shelterin**				
TRF1	Increase [19,30]	Increase [30]		
TRF2	No Change [19]	No Change ^d^ [31]		
POT1	Increase [32]	Increase [33]		
**Helicase**				
BLM	Increase [19]	OE Increases [31]	Decrease [34]	
WRN	No Change [19]			
RTEL1	Increase ^b^ [6]	Decrease [35]		
RECQL4	Increase ^b^ [36]			
**Nuclease**				
DNA2	Increase ^b^ [37]			
MRE11	Increase [38]	Decrease [21]		
SLX1/SLX4	Increase [34,39] Decrease ^c^ [25]	Decrease [20]	No Change [34]	
MUS81	No Change ^c^ [25]	No Change [20]	No Change [34]	
XPF	Increase ^c^ [25]		Decrease [40]	
Apollo	Increase [41]			
**RNA Metabolism**				
RNaseH1	Increase [21]	Increase [21]		
NONO	Increase, Leading Strand [42]			
hnRNP1A	Increase [43]			
**HR Factor**				
BRCA1	Increase: U2OS [44] No Change: MEF [45]	Increase [21]		
BRCA2	Increase ^b^ [38,45,46]	Increase [38]		
RAD51	Increase [21]	Increase [21]	Increase [16]	Increase [16]
RAD51AP1	Increase or Decrease ^f^ [25,47]			Decrease [47]
RAD52	No Change [21]	Decrease [21]	Decrease [34]	Increase [34]
**Fork Remodeler** **/Protector**				
FANCD2	Increase [48,49]			
FANCM	OE Decreases [50]		Increase [40,51]	
Timeless/Tipin	Increase [52]	Increase [21]		
STN1/CTC1	Increase [22,27,32,53]			
SMARCAL1	Increase [54]			
ZRANB3	Decrease ^g^ [29]			
PARP1	Decrease ^g^ [29]			
**DNA Replication Factor**				
POLD1,3,4	No Change TRF1ko [30], Decrease ^c^ [25]	Decrease [30]	Decrease [16]	Decrease [16]
POLE1,2			Increase [16]	Increase [16]
POLA1,2			Increase [16]	Increase [16]
Prim1				No Change [16]
MCM2/7				Increase [16]
PCNA			Decrease [16]	Decrease [16]
RFC1			Decrease [16]	Decrease [16]
POLH (Pol eta)	Increase With APH [55]	Increase With APH [55]		No Change [16]
REV3L (Pol zeta)			Increase [16]	Increase [16]
FEN1	Increase ^b^ [56,57]			
**DNA Excision-repair Protein**				
OGG1	Increase ^e^ [24]	Increase ^e^ [24]		
CSB	Increase ^b^ [54]			
MSH6	Increase [58]			
MSH2	Decrease [59]			
MSH3	Increase [59]			
XPC	Increase [28]			
XPD, XPB, TFIIH	Increase [60]			
**Mitotic Checkpoint**				
BUB1/BUB3	Increase [61]			
Aurora Kinase B	Increase [62]			
**Chromatin Structure and Cohesion**				
TopIIα	Increase [63]			
SMC5/6	No change [30]	Decrease (ALT) [21], No change (TRF1ko) [30]		
INO80	Increase [64]			

* Knockdowns or knockouts unless noted with OE (over expressed). ^a^ OE = over expressed. ^b^ Additive with aphidicolin. ^c^ BLM knockout MEFs. ^d^ When over expressed (OE) with SUMO conjugated, MiDAS increases. Mutants that cannot be SUMOylated decrease MiDAS [31]. ^e^ With FAP-TRF1 induced 8oxoG. ^f^ Increase with siRNA and decrease with sgRNA in U2OS. ^g^ RTEL1 knockout cells.

## Data Availability

Not applicable.
